# How three different theories of depression converge at inflammation

**DOI:** 10.1007/s44192-025-00312-4

**Published:** 2025-12-24

**Authors:** B. Baumberger, L. Batey, P. Hashemi

**Affiliations:** Department of Bioengineering, Imperial College London, London, SW7 2AZ UK

## Abstract

Three main theories of depression have been developed: the monoamine, plasticity and inflammation theories. While each theory has been bolstered by decades of excellent scientific evidence, individually each idea falls short for developing universally effective treatments for depression. In this perspective, we present the history and development of each theory and discuss the therapies that follow each hypothesis. We provide a unique perspective by highlighting the historical evolution, clinical implications, and the nexus between the three hypotheses. We emphasise how the theories are mutually inclusive and influence one another and are smaller parts of a larger puzzle. We suggest that future therapies should involve all three: ze: monoamines, plasticity and inflammation.

## Introduction

Human health has improved in many ways over the last century; however, the understanding and management of mental health continues to lag. Incidences of mental health disorders are continually rising, with an estimated 300 million people diagnosed globally with depression alone. This number (which is likely an underestimate due to stigma and lack of access to healthcare) renders depression one of the globe’s most prevalent disorders.

Diagnosis and treatment of depression are inconsistent and controversial because the underlying pathology of this illness has yet to be fully elucidated. There have been three major theories of depression: the monoamine, plasticity and inflammation theories. There is compelling evidence for each theory, but individually they fail to fully explain the phenotype and universally treat the symptoms of depression. In this perspective, we will cover the development of these major theories and discuss their associated therapies. We speculate that these theories are not discrete but rather form small and synergistic pieces of the larger puzzle of depression pathology, around the central theme of inflammation. Finally, we suggest that future therapies should be designed around all three aspects: monoamines, plasticity and inflammation.

### Theories of depression

Until the 1950s, depression was so ill-understood that treatments were arbitrary and, at times, cruel. Thousands of years ago, depression was a spiritual issue, and treatments involved trephinations [1]. In 600 BC the ancient Greeks employed bloodletting and purging to restore balance to the humors of the body [2]. Post 1600s, depressed individuals were institutionalized and isolated from other people, treated with ice baths and restraint techniques [3]. There are scattered reports in history of using opiates and barbiturates for melancholy [4] and manic-depressive psychosis [5], however more progress was made in the early 1900s, when the world entered a modern age of medicine that included important drug discoveries, including insulin [6]. Insulin coma therapy was the first time drugs were used on a large-scale for depression. Here, patients were forced into a hypoglycemic coma under the rationale that this state would “reset” the brain [7]. Around the same time, Metrazol, a nervous system stimulant, was used to induce seizures to the same effect [8]. This precipitated the highly controversial electroconvulsive therapy (ECT) in the 1940s as an alternative to Metrazol because it enabled more precise control of the duration and intensity of the seizures [9]. Also during this time, amphetamines were being explored for their effects on alleviating depression [10] becoming incredibly popular in the 1950s as medications for mild depression after strong marketing campaigns from Smith, Kline and French [10]. However, it became quickly apparent that these agents were addictive [11], patients became tolerant, and withdrawal itself induced depression [10]. Thus, in 1971, amphetamines were declared a Schedule-II controlled substance and no longer used for depression [12]. Perhaps the most infamous depression treatment was the lobotomy, which was first introduced around the same time. A celebrated therapy at this time (Egas Moniz won the Nobel Prize in Medicine for the lobotomy in 1949) [13], this treatment was later recognized as unethical and harmful. These early 20th century treatments became obsolete in the 1950s, when chemical theories of depression began to be developed.

### Monoamine theory of depression

*“Depression is caused by a deficiency of serotonin*, * dopamine and norepinephrine” Mid 1960s.*

The 1950s were a time of rapid growth for biochemistry and drug development and transformed how the scientific and medical communities viewed depression. Scientists at Hoffmann La Roche discovered interesting ‘side effects’ of a new compound they were trialing for tuberculosis [14]. These side effects included ‘euphoria’, improvements in sleep and better appetite. This drug, iproniazid, kills bacteria by inhibiting the synthesis of mycolic acid, a key component of the bacterial cell wall, but this drug is also a monoamine oxidase inhibitor (MAOI). Monoamine oxidase is found at high concentrations in the brain and is the key enzyme in the brain that is tasked with catabolizing monoamine transmitters (serotonin, dopamine and norepinephrine). Iproniazid was the first dedicated antidepressant and was used off-label to treat depression in 1958. At the same time, scientists started to investigate the roles of neurotransmitters in depression to develop more selective drugs.

Imipramine, first synthesized by Hafliger and Schindler [15], was originally investigated as an antipsychotic treatment. This agent did not have anti-psychotic effects in patients but was found to improve depression symptoms [16, 17]. Imipramine was approved by the FDA to treat depression in 1959. It is a tricyclic antidepressant (TCA), which inhibits the reuptake of norepinephrine and serotonin back into neurons [18]. 

Seminal studies in the mid-1960s found that serotonin levels were lower in the brain of depressed suicide patients [19], and norepinephrine levels were reduced in the cerebrospinal fluid and urine of depression patients [20]. These findings, taken together with the pharmacological success of MAOIs and TCAs, propelled the development of a new theory of depression. This new theory, the monoamine hypothesis of depression [21], postulates that depression is caused by a deficiency of serotonin, dopamine and norepinephrine. As such, Eli Lilly started developing agents to selectively inhibit serotonin reuptake *via* the serotonin transporters, under the rationale that this effect would correct a serotonin deficiency. In 1974, the first paper describing a selective serotonin reuptake inhibitor (SSRI) was published [22, 23], the agent was named LY110140 or fluoxetine and approved by the FDA and marketed as Prozac ^(^™^)^ in 1987 [24]. 

A rich and varied body of experimental literature has studied monoamine roles in depressive disorder [25–30]. While it is not yet possible to measure human brain monoamines as a biomarker for depression, exciting recent work in humans using positron emission tomography showed that the release of serotonin was reduced in depressed vs. control patients [31]. Thus, this might represent an exciting new direction for biomarker research. Since Prozac^(^™^)^, an array of serotonin, norepinephrine and dopamine targeting agents have been approved (and some taken off the market) for depression [32, 33]. Of these, a small subset is routinely prescribed to vast numbers of people. These agents have helped millions of people and represent huge advances in depression treatment over the last century. However, in recent years, the variable success rate and delayed onset (patients typically must take SSRIs for weeks for clinical effectiveness) of therapeutic actions of SSRIs have pointed towards additional mechanisms outside of monoamines and have thus spurred the medical community to seek alternative theories of depression.

### Neuroplasticity and neurogenesis

*“Depression is caused by impairments in the brain’s ability to adapt and generate new neurons and neuronal connections” Mid 1990s*.

A healthy brain can reorganize itself by forming new neural connections. This process, called neuroplasticity, allows the brain to learn new information, recover from damage and adapt in response to new experiences and changes in the environment. In two specific brain regions, the hippocampus and the subventricular zone, the brain can also actually regenerate itself, in a process called neurogenesis [34, 35]. 

The hippocampus became a critical area of interest in depression studies in the mid-1990s because scientists found, utilizing volumetric MRI, loss of hippocampal volume in a group of older women with recurrent major depression [36]. The same group confirmed their original report in a later study and showed a correlation between hippocampal volume and the duration of depression [37]. Other work during this period found a similar loss of hippocampal volume in post-traumatic stress disorder patients [38]. These clinical findings were supported by a series of animal studies earlier that decade, which found that acute stress suppressed the rate of dentate gyrus (part of the hippocampal formation) neurogenesis in adults of several species [39]. 

The plasticity hypothesis of depression was solidified in a seminal Society for Neuroscience Meeting in 1999 where Barry Jacobs and his colleagues presented a paper entitled “Chronic Fluoxetine Treatment Increases Hippocampal Neurogenesis in Rats: A Novel Theory of Depression”. Here, they showed that chronic fluoxetine increased hippocampal neurogenesis in rats (70% increase in the number of cells) [40]. Other work supported this finding into the 2000s [41, 42], and eventually some evidence pointed towards the same in humans [43, 44]. However, hippocampal volume was not successful as a biomarker for depression because of individual variability in hippocampal size and overlap with other illnesses [45–47]. 

The proposed mechanism for the neuroplasticity theory is that stress and depression induce glucocorticoids (stress hormones) which decrease glutamate [48] and serotonin levels [49–51]. Decreased glutamate can cause widespread morphological changes in the hippocampus [52]. Serotonin is a well-known neuroplastic factor [53], however the inherent question here is can glutamate itself be pharmacologically targeted for depression?

#### Ketamine

Ketamine is an NMDA (glutamate) receptor antagonist [54] which caused much excitement as a potential antidepressant in 2000 with the publication of its first double-blind placebo-controlled trial for major depression [55]. A contributing factor to the buzz around ketamine was the rapidity of onset as an antidepressant. Whereas SSRIs typically need to be administered for several weeks for clinical effects, several studies have shown that antidepressant effects could be achieved within hours or days of a single subanesthetic dose of ketamine given intravenously [55–59]. 

**Fig. 1:**
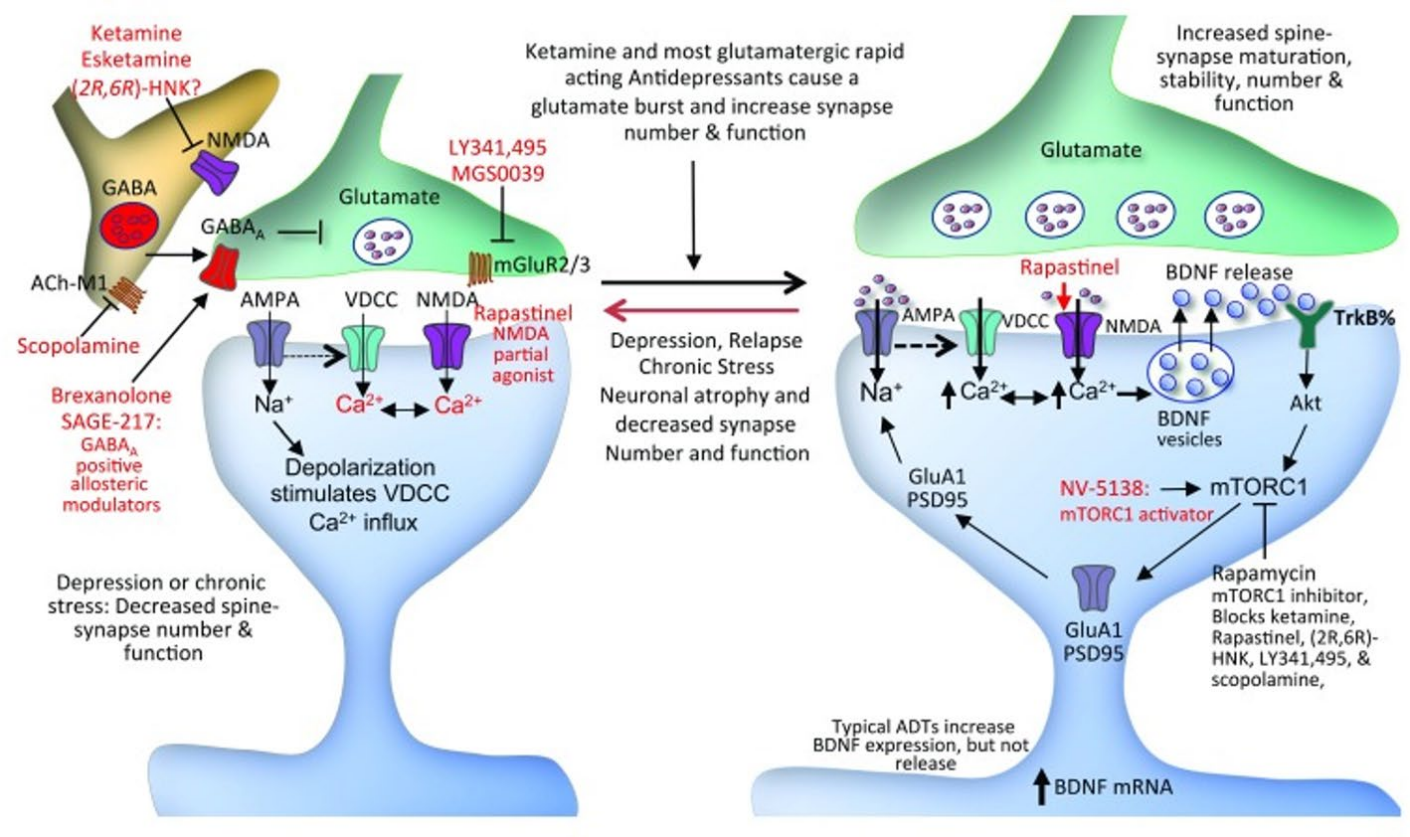
Mechanism of ketamine action on plasticity, taken from Duman [63].

The mechanisms for this rapid onset of action are still being studied [60, 61], but it has been found that a single ketamine dose rapidly increases the number and function of synapses. This plasticity is thought to arise from a surge of glutamate that causes the release of brain-derived neurotrophic factor (BDNF), activation of mechanistic target of rapamycin complex 1 (mTORC1) [62] and tropomyosin-related kinase receptor B - protein kinase B (TrkB-Akt) signalling, and the rapid synthesis of synaptic proteins that contribute to the formation of new synapses (see Fig. 1) [62–64]. 

While the effects of ketamine on plasticity are consistent, one study found that the antidepressant effects of ketamine are independent of the effects on neuroplasticity, implying other nuances in the mechanisms of action [65]. In addition, ketamine’s effectiveness as an antidepressant is variable and very difficult to compare to SSRIs. This is because typically, only patients who do not respond to SSRIs undergo ketamine therapy. Moreover, there are ethical and safety concerns around ketamine because of its potential for abuse and its toxicity [66–68]. 

As ketamine was being explored for its antidepressant effects, a parallel new idea was brewing. It is well known that psychedelics are neuroplastic agents [69], and after some clinical success using psychedelics to treat obsessive compulsive disorder [70] and tobacco [71] and alcohol addiction [72], a natural new direction was to explore psychedelics in depression.

#### Psychedelics

Psychedelics, such as psilocybin, are powerful serotonergic hallucinogens that have long been known to alter perception and affect cognitive processes [73]. Importantly, these agents are known to affect mood [73]. There were clinical studies in the mid-2010s, using small patient cohorts (10–20 patients per study), that found promise in psychedelic therapy for recurrent or treatment resistant depression [74–77]. A key feature of these studies is that the psychedelic was administered in conjunction with psychotherapy. Psychotherapy is known to change brain function [78] in a way that suggests plasticity is involved [79, 80]. One notion is that the connections associated with painful memories/stress/trauma are gradually replaced and/or overwritten with a larger number of connections that cue more positive emotions.

Therefore, one way to integrate psychotherapy with psychedelic therapy is to suppose that serotonergic psychedelics render the effect of psychotherapy more effective and efficient. Indeed, it was hypothesized by Carhart Harris et al. that the therapeutic effects of psychedelics are “*fundamentally reliant on context”* [81]. Hence, providing psychological support and a positive environment (good atmosphere) is a key component for clinical success. Not providing the correct environment or support means that the treatment may not only be clinically ineffective but also potentially harmful [82]. As such, the state-of-the-art psychedelic treatment involves the patients being treated with psilocybin in a custom-designed treatment room with low lighting, music and full support from two councilors at all times [83]. The psychotherapy includes preparation and integration sessions which taken together constitutes a considerable amount of time. The neuroplastic effects of the psychedelics, therefore, are very much guided by a positive environment and psychological guidance.

In sum, ketamine and psychedelics are exciting avenues of research for depression that are being actively trialed and explored. There are, however, concerns that both are controlled substances with abuse potential and limited effectiveness. Therefore, the community is eagerly anticipating the results of large-scale trials for drug efficacy and safety.

### Cytokine/inflammation theory of depression

*“Depression is caused by immune activation” Early 1990s*.

In parallel to the plasticity theory of depression, another hypothesis was being developed, based on inflammation. In 1991, Ronald Smith, professor of Chemistry and Nutrition at Gavilan College, published a new hypothesis of depression in a paper entitled “The Macrophage Theory of Depression” [84]. In the work, he argued that cytokine secretion was the cause of depression under several points of rationale, but most importantly, human volunteers that were treated with cytokines developed depressive-like behaviors [85]. This notion was quickly backed up in 2 papers from Maes et al., who suggested that sickness behavior and depression share common pathways in the form of immune activation [86, 87]. Since these early papers, a superfluity of studies have linked inflammation to depression, with a comprehensive summary of the literature provided in a review by Nemeroff and colleagues [88].

**Fig. 2:**
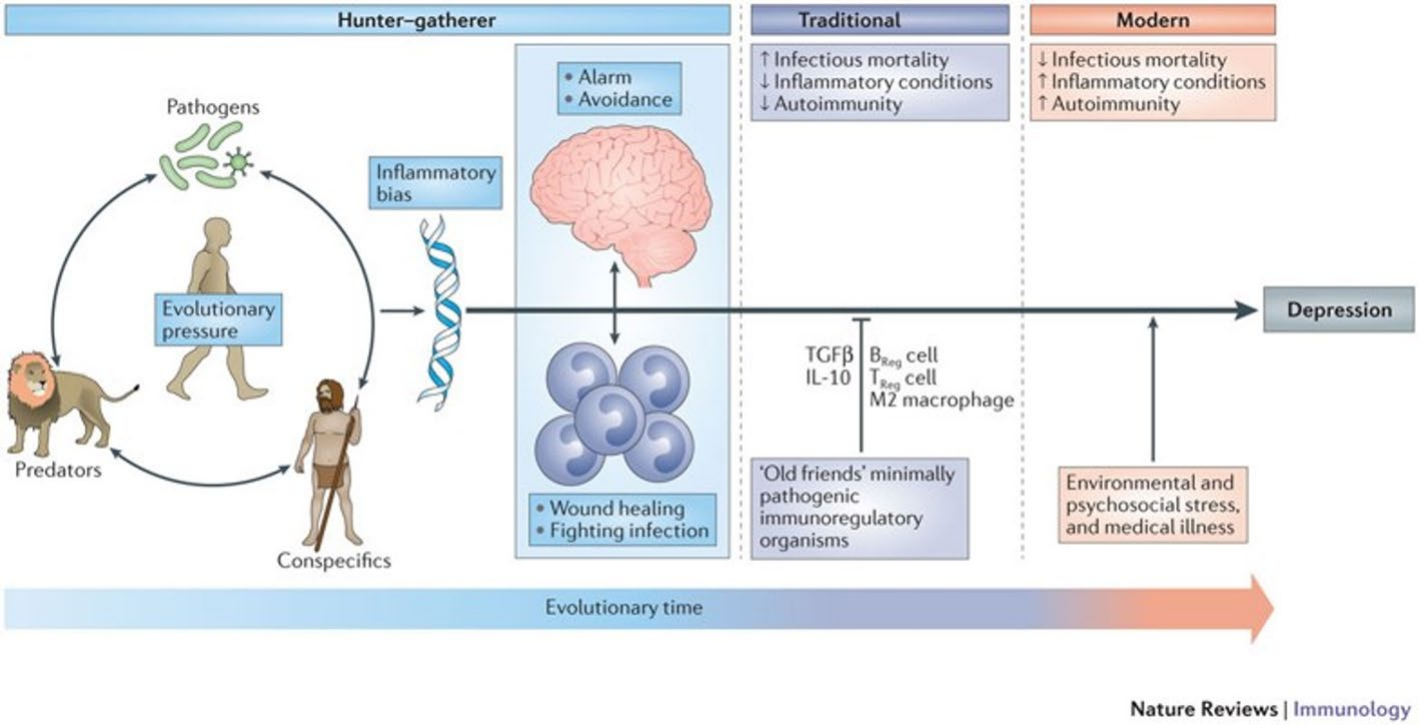
Evolutionary hypothesis of inflammation and depression, taken from Miller et al. [103]

Inflammation is a general term we use here to include the dysfunction of hypothalamic-pituitary-adrenal axis (HPA) gut-brain axis and endocrine systems. It is essentially undisputed that chronic illnesses and depression are comorbid and the precise mechanisms of how inflammation drives behavior are still being elucidated. Chronic illnesses such as chronic stress [89–91], hypertension [92, 93], and HIV [94] carry with them a greater rate of depression diagnosis compared to the general population. Recent findings from the COVID pandemic further bolster the connection between inflammation (in this case acute inflammation triggering a storm of pro-inflammatory cytokines such as interleukin-6 (IL-6), tumor necrosis factor-alphaTNF-α), and interleukin-1 betaIL-1β)) and depression [95, 96]. This mechanism is clearly considerably complicated involving blood brain barrier function [97] and overproduction of these cytokines [98]. Cytokines have been shown to directly affect brain function by altering neurotransmitters [99, 100] in particular the mood driving serotonin, dopamine, and norepinephrine. The effects of inflammation on these monoamine levels in the brain has recently been reviewed by Batey et al. [101] Lipopolysaccharide (LPS)—induced inflammation, through varied and nuanced mechanisms involving the blood brain barrier [102], reduces the brain’s serotonin and dopamine signaling and increases norepinephrine signaling [101]. This effect on mood-altering neurotransmitters begs the question, why does inflammation induce depressive behavior? Early evolutionary immune pressures were primarily pathogenic in nature and the mood response was directed towards energy conservation for healing and recovery. In modern society, where pathogens are less of a challenge, social issues (e.g. stress) have become the predominant immune activator, as seen in Fig. 2. The latter is less tangibly resolved and can often be chronic [103]. 

The effect on inflammation on neuroplasticity is also increasingly being appreciated. Researchers have found that inflammatory conditions including chronic stress [104], stroke [105], traumatic brain injury [106] and multiple sclerosis [107] coincide with plasticity changes. The link between inflammation and plasticity is likely microglial BDNF mediated [108, 109]. 

**Fig. 3:**
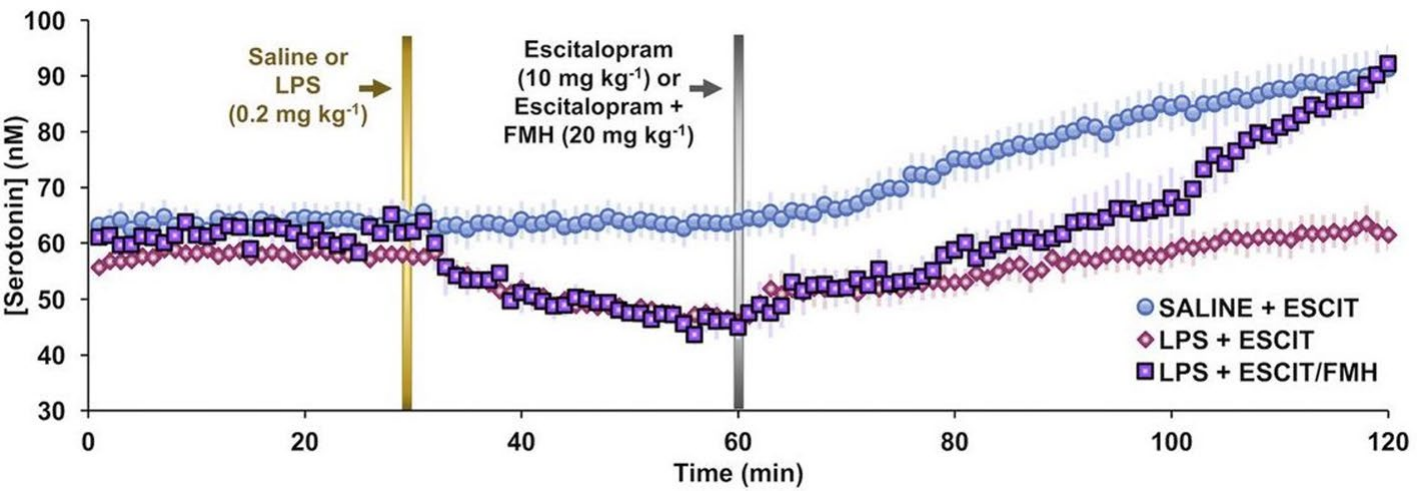
Peripheral LPS effects on brain serotonin. Taken and modified from Hersey et al. [111]

There is compelling evidence to show that patients with high levels of inflammation are more likely to be resistant to conventional SSRI treatment [110]. In animal work, SSRIs were shown to be less capable of restoring serotonin levels under inflammation. This was due to inflammation-induced brain histamine which inhibits serotonin *via* H_3_ receptors [111]. Figure 3 shows basal serotonin measurements in the mouse hippocampus. Two cohorts (red and purple) received peripheral LPS and very rapidly the serotonin levels declined. Serotonin levels could not be recovered to control values with escitalopram alone (red). The co-application of an agent that inhibited histamine synthesis (purple) was necessary to resolve the serotonin levels to control values. Therefore, utilizing anti-inflammatory therapies may represent an exciting new avenue for depression.

The literature on using anti-inflammatories as a depression treatment is mixed but shows some promise [112–115]. Non-steroidal anti-inflammatory drugs (NSAIDs) and cytokine inhibitors are the main therapies used [116, 117], but there are also many adjuncts such as statins [113]. Interestingly, ketamine and psychedelics are also considered to have anti-inflammatory properties [118, 119].

A challenge with this anti-inflammatory treatment is dosage. Recent reviews of the clinical literature that correlate markers of inflammation to depression concluded that inflammatory responses in depressed patients were different from control patients in approximately one-third of subjects (similar to SSRIs). The studies reviewed disagreed on the direction and the magnitude of the differences [113, 114, 120]. This is an extremely important aspect to decode when trying to treat depression with anti-inflammatories since there is evidence showing that using anti-inflammatory agents in patients without inflammation can lead to adverse outcomes [121].

In sum, the inflammation theory of depression describes a potential behavioral outcome of the evolutionary response to acute and chronic illnesses and to modern stressors. Anti-inflammatories serve as an exciting new therapy for the treatment of depression with the remaining challenges for type, dose and side effects.

## Three theories: one story

**Fig. 4:**
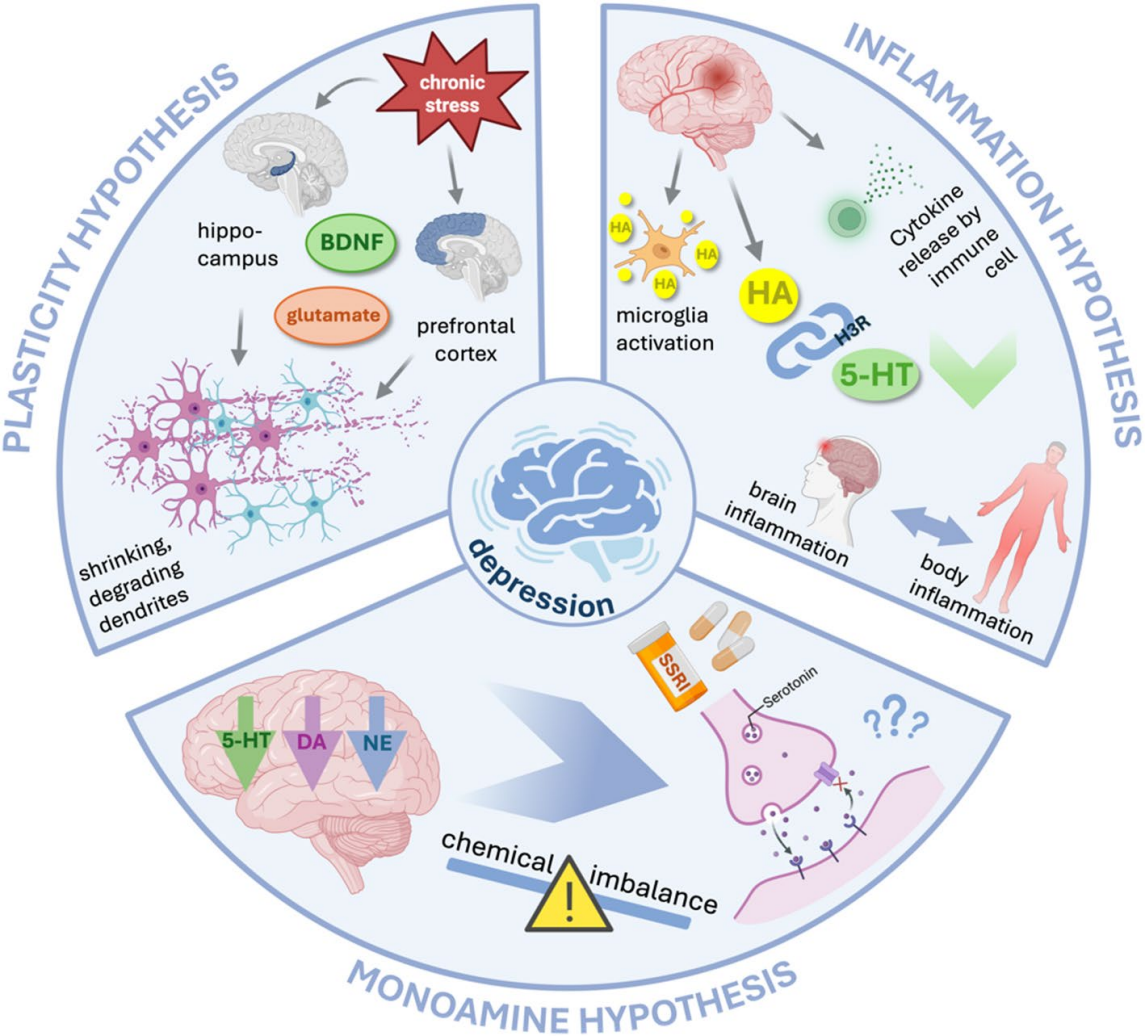
Diagram of three theories of depression: plasticity, monoamine and inflammation. Figure created with BioRender

It is clear to see, now, that the three main theories of depression are small parts of the same jigsaw puzzle (Fig. 4). During inflammation, the body has evolved to conserve energy to recover, thus, mood-driving monoamine levels change to achieve this outcome. While the sickness behavior serves its purpose evolutionarily, in modern sanitized societies, the response is synonymous with stress. Low serotonin is not a cause but a consequence of depression and can serve as an indicator to diagnose and monitor depression progression. Targeting serotonin does not necessarily help patients recover from depression, since the cause of depression often lies in inflammation rather than a singular marker in serotonin. This issue leaves the majority of depressed patients (with differing levels of inflammation) partially or totally unresponsive to SSRIs.

This lower serotonin, along with lower glutamate and other facets of inflammation, decreases hippocampal plasticity which can be recovered to differing extents with SSRIs, ketamine and psychedelics, though none are universally efficacious. Again, this is because the loss of neuronal connections is a result (marker) and not the cause of depression.

The issue of targeting inflammation is complicated. A big challenge is creating a robust system to measure neuroinflammation as a biomarker of depression. Without this, we don’t know how and when to intervene in the inflammation chain to stop the detrimental effects on mood.

To conclude, we propose that the monoamine, plasticity and inflammation theories of depression are not discrete or in competition with each other. They are, at their core, foundational parts of a larger story. Therefore, improving future treatments is critically dependent on accurately and chemically defining ‘neuroinflammation’ status and considering all three theories for drug discovery.

## Data Availability

No datasets were generated or analysed during the current study.
